# Nosewitness Identification: Effects of Lineup Size and Retention Interval

**DOI:** 10.3389/fpsyg.2016.00713

**Published:** 2016-05-30

**Authors:** Laura Alho, Sandra C. Soares, Liliana P. Costa, Elisa Pinto, Jacqueline H. T. Ferreira, Kimmo Sorjonen, Carlos F. Silva, Mats J. Olsson

**Affiliations:** ^1^Department of Education and Psychology, University of Aveiro, AveiroPortugal; ^2^Institute for Biomedical Imaging and Life Sciences (IBILI), Faculty of Medicine, University of Coimbra, CoimbraPortugal; ^3^Center for Health Technology and Services Research (CINTESIS.UA), Faculty of Medicine, University of Porto, PortoPortugal; ^4^Department of Clinical Neuroscience, Division of Psychology, Karolinska Institutet, StockholmSweden

**Keywords:** nosewitness, forensic psychology, lineup identification, lineup size, retention interval

## Abstract

Although canine identification of body odor (BO) has been widely used as forensic evidence, the concept of *nosewitness identification* by human observers was only recently put to the test. The results indicated that BOs associated with male characters in authentic crime videos could later be identified in BO lineup tests well above chance. To further evaluate nosewitness memory, we assessed the effects of lineup size (Experiment 1) and retention interval (Experiment 2), using a forced-choice memory test. The results showed that nosewitness identification works for all lineup sizes (3, 5, and 8 BOs), but that larger lineups compromise identification performance in similarity to observations from eye- and earwitness studies. Also in line with previous eye- and earwitness studies, but in disagreement with some studies on odor memory, Experiment 2 showed significant forgetting between shorter retention intervals (15 min) and longer retention intervals (1-week) using lineups of five BOs. Altogether this study shows that identification of BO in a forensic setting is possible and has limits and characteristics in line with witness identification through other sensory modalities.

## Introduction

Witnesses have an important role in criminal processes (e.g., Ashworth and Redmayne, 2010), especially in the absence of any other type of evidence (Odinot and Wolters, 2006). The identification of perpetrators has typically been made by eyewitnesses (e.g., Wells and Olson, 2003), but to some extent also by earwitnesses (Yarmey, 1994; Hollien, 2012; Hollien et al., 2014).

Although identification of culprit body odor (BO) has also been used as evidence in court in many countries (e.g., Prada and Furton, 2008; Ensminger et al., 2010), these identifications have typically been made by dogs and not by humans (Stockham et al., 2004; Schoon, 2005). It is a common belief that human olfaction is inferior to other mammals. Although olfactory acuity can be operationalized in different ways, it is interesting to note that a recent comparative review of absolute thresholds for a number of monomolecular substances shows that humans outperform many other mammals although not for the dog (Laska, in press). In line with this, dogs have been shown to successfully match human odor samples to individuals (Syrotuck, 1977; O’Block et al., 1979). A number of studies have shown that the performance levels of dogs are typically in the range of 75–90% correct (Settle et al., 1994; Schoon, 1996; Marchal et al., 2016). Only one study so far has investigated human identification of BO in a forensic set up (Alho et al., 2015; see below).

Since witnessing a crime may very well be from the perspective of a victim in close interaction with an offender, the question arises why olfaction has been virtually absent in the forensic field. In fact, humans show the ability to discriminate others’ BO from their own (e.g., Platek et al., 2001). They can also use odor to tell their relatives (e.g., Lenochova and Havlicek, 2008), friends, and unrelated individuals apart from each other (e.g., Olsson et al., 2006). A recent study indicated that odors of different individuals unknown to the participant could be discriminated well above chance level (Allen et al., 2015). In addition, reports indicate that victims sometimes do remember the BO of their offenders (e.g., Christianson, 1992; Doege, 1992). This might be particularly relevant in crimes such as sexual aggression or physical assaults where the victim and the offender are close together and in particular if visual inspection is compromised by e.g., darkness or blindfolding.

Pertinent to odor identification in forensics, each individual has a unique “odor print” that is genetically determined (e.g., Beauchamp and Yamazaki, 2005; Penn et al., 2007; Rodriguez-Lujan et al., 2013) and that stays fairly stable over time (e.g., Schoon, 1996; Roberts et al., 2013), although other factors like diet, health and aging may modify that specific odor to some degree (Havlicek and Lenochova, 2006; Mitro et al., 2012; Olsson et al., 2014). In two experiments, Alho et al. (2015) tested episodic recognition memory for BOs in a forensic setting. In the first experiment, the authors introduced the *nosewitness* paradigm, using a target-present (e.g., the culprit was always present in the lineup), forced-choice lineup test in an emotional and a neutral condition. The results indicated that BOs associated with male characters in authentic crime videos could later be identified in BO lineup tests well above chance, on par with reports on eyewitness identification. In the second experiment, these findings were replicated by following the standard procedures in the administration of lineups (e.g., target-present and target-absent lineups). Whereas performance on target-present trials was highly significant, on target-absent trials performance approached chance level.

Given the promise of this first study, we decided to further investigate the notion of nosewitness identification, particularly how olfactory memory is affected by some factors. In two experiments, using a nosewitness condition (in which an emotional crime video is presented together with a BO), we explored two variables that have effects on eyewitness identification performance: lineup size and retention interval (RI). The lineup procedure is characterized as a *system variable* (Wells, 1978; Wells and Olson, 2003). This means that the lineup structure can be controlled by the criminal justice system. The size of the lineup is an important factor since it influences the accuracy of the witness (Leach et al., 2009). Working memory, which involves the short-term maintenance of information and is required in many cognitive tasks (Dade et al., 2001), may be more challenged in larger lineups than in shorter ones.

The RI – that is, the amount of time between the crime and the presentation of a lineup to the witnesses – is an important *estimator variable* to consider in lineup identification of criminals (Wells and Loftus, 2003; Deffenbacher et al., 2008; Paz-Alonso and Goodman, 2008; Ahola, 2012). Contrary to the system variables, the estimator variables cannot be controlled by the justice system (e.g., Wells and Olson, 2003). The study of forgetting rate is of particular interest, since the time from witnessing the crime to identifying a perpetrator can vary from hours to months or even years (Sauer et al., 2010). Although some studies have shown remarkably little forgetting in episodic recognition of odors compared to other modalities, most recent investigations demonstrate impaired recognition with longer RIs (Cornell Kärnekull et al., 2015).

## Experiment 1

In Experiment 1, we probed identification of BO in a forced-choice (target present) lineup memory test, manipulating the lineup size. The forced-choice procedure was chosen for three reasons. First, the procedure targets memory capacity free from decision bias. Second, a previous study (Alho et al., 2015, Experiment 2) employing the target-absent/target-present procedure showed that correct rejections for target-absent lineups were close to chance levels indicating that this type of lineup for the current purpose is fraught with floor effects, which makes the assessment of memory performance insensitive for experimental manipulations. Thirdly, since this study targeted the effect of lineup position, lineups with target presents (i.e., forced-choice) is preferred.

In the witness session, an authentic video-clip of a violent crime was presented along with a BO. Written instructions prompted the participant into the mindset of an eyewitness to make the experimental model of the nosewitness situation more realistic. The cover story stated that the BO was coming from the male character (the culprit). In a later lineup test, participants decided which BO sample out of 3, 5, or 8 total samples was the culprit’s. Since odors may be hard to discriminate (Olsson and Cain, 2000; Allen et al., 2015), are subject to sensory adaptation (Ekman et al., 1967) and are a challenge for working memory (Andrade and Donaldson, 2007), especially when lacking a supportive verbal label (Jönsson et al., 2011), we hypothesized that larger lineup sizes would compromise identification performance more than smaller ones.

### Method

Both experiments were approved by the Ethics Committee of the University of Aveiro, Portugal. Moreover, the guidelines of the Declaration of Helsinki and the standards of the American Psychological Association were followed.

#### Body Odor Samples

Body odor samples were collected from the armpits of 51 healthy male students from the University of Aveiro, aged between 18 and 28 years (*M* = 21.57, *SD* = 2.24), while they were in class (non-stressful period). Donors were male (consonant with the fact that a vast majority of criminals are men; Kanazawa, 2009), non-smoking, medication free and without any physical, metabolic or mental disease. Donors were instructed to refrain from using fragrant hygiene products, drinking alcohol, eating spicy foods and performing any activity that would alter their natural BO starting 24 h before the sampling until the moment they came back to the lab to deliver the BO samples.

Body odor was collected on nursing pads (Mimo Natura, Portugal) sewn into the armpits of t-shirts previously washed with odorless detergent (as recommended by, e.g., Mitro et al., 2012). Participants wore the t-shirts in a campus lecture room for 4 h. The nursing pads were then collected from each armpit, divided into equal size quadrants, put in a zip-locked bag and frozen at -20°C.

The pad quadrants were thawed 1 h before testing. Two pad quadrants were placed along the walls of wide-mouthed glass jars with lids and were used as BO samples. To prevent contamination, odor samples were always handled with surgical gloves.

#### Participants

Seventy-three students (36 men aged between 18 and 30 years, *M* = 22.39, *SD* = 2.97, and 37 women aged 18–33 years, *M* = 21.89, *SD* = 3.21) from the University of Aveiro volunteered to participate. The participants did not suffer from any mental, neurological, metabolic, or respiratory diseases and were medication free. They were asked to refrain from eating (e.g., gum, candies, mints), drinking coffee, or using any products that could interfere with their ability to smell 1 h before testing (e.g., perfumes). Participants and donors signed a written informed consent form, including the right to abort participation at any time, and when applicable were rewarded with course credits.

#### Design and Procedure

In a between-subject design, participants were randomly assigned to one of the three conditions: lineups with three BOs (*n* = 24, 12 males), lineups with five BOs (*n* = 25, 12 males), and lineups with eight BOs (*n* = 24, 12 males), in which they viewed a 1-min audio-visual presentation (video clip) of a crime involving a man (culprit) and a woman. Instructions were displayed on the screen for 14 s stating: “You will see a real crime captured by a video camera. During the video you will be exposed to an odor collected from the perpetrator of the crime you will be watching.”

Participants either witnessed a sexual assault (video #1) or a theft with a hostage taking (video #2). These two crime videos were selected from Alho et al. (2015) for being rated as highly vivid and arousing. Both videos were presented on a 17′′ computer screen with an approximate viewing distance of 50 cm. Participants used headphones. During the video clip, a BO was presented continuously from a wide-mouth glass jar. Participants were instructed to breathe through their nose.

In a 15-min period between the video clip (witness session) and lineup test, participants rated the video on a 9-point scale in terms of vividness (1 being *not vivid* and 9 being *very vivid*), pleasantness (1 being *very unpleasant*, 5 being *neutral*, and 9 being *very pleasant*) and arousal (1 being *not arousing* and 9 being *very arousing*) and completed a questionnaire assessing trait anxiety (STAI-T, Silva and Spielberger, 2007).

In the lineup test, participants were instructed to identify the odor of the culprit who’s BO they smelled during the video presentation. Participants had to choose from a lineup of 3, 5, or 8 BO samples (one culprit and foils). This forced-choice target-present procedure was chosen in order to obtain a high power and bias-free measure of identification performance. BO samples were presented in wide-mouth glass jars, from left to right, with no time restriction to smell the BO, but without the chance to resample previous BOs. The full instructions stated: “You have a lineup with three/five/eight different BOs in front of you and you will smell all of them from left to right. The BO that you smelled during the video is present in this lineup. You can smell each BO as long as you wish, but you can’t go back and re-smell them. Make a pause of 6 s between each BO. After you smell all of the BOs, indicate your identification response on the sheet, please.”

The counterbalancing of the BO samples was arranged in order to assure that the culprit BO was presented in each of the positions of the lineup and that the samples were thawed and refrozen the same number of times within conditions. Due to the number of samples being different between conditions (3, 5, and 8 BO lineups), and since we had two crime videos, the counterbalancing of the BOs was made taking into account the two videos (i.e., the same lineups were used in the presentation of the video #1 and in the presentation of video #2). Thus, we used new sets of odors in each lineup size condition and although the targets were not used as foils, we used different target-BOs within conditions. The interstimulus-interval of 6 s was chosen in an attempt to balance constraints of odor adaptation and working memory capacity (Jönsson et al., 2011).

After making their identification, participants were asked to rate their confidence in their decision on a scale from 0 to 100%. Finally, participants were thanked and received further information about the nature of the experiment.

Both before and after the task, participants rated their perceived stress using a 100 mm visual analog scale, from *not stressed at all* to *very much stressed*, and their state anxiety levels (STAI-S, Silva and Spielberger, 2007). The purpose was to monitor whether participants were in distress when they finished the experimental task, as well as to assess whether any of these measures was correlated with performance.

### Results and Discussion

#### Nosewitness Experience

The ratings of the crime videos clearly indicated that the crimes were experienced as highly vivid (*M* = 6.47, *SD* = 1.93; *M* = 6.49, *SD* = 1.47, for video #1 and #2, respectively), arousing (*M* = 6.17, *SD* = 1.81; *M* = 6.65, *SD* = 1.74, for video #1 and #2, respectively) and unpleasant (*M* = 2.14, *SD* = 1.53; *M* = 1.78, *SD* = 1.25, for video #1 and #2, respectively). Independent samples *t-*tests of the crime video ratings indicated no statistically significant difference in the evaluation of the two videos (*p* > 0.05). They will therefore not be a factor in the following analyses.

#### Lineup Identification Performance

**FIGURE 1:**
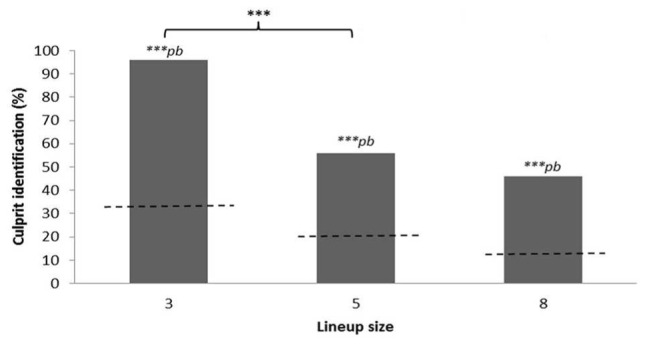
**Percentage of participants correctly identifying the culprit odor in the three lineup size conditions.** Dashed lines in the bars represent chance levels for each lineup size condition (33.3, 20, and 12.5%, respectively). Asterisks refer to a significant chance-level corrected difference (odd ratios analyses) between the conditions 3 and 5 (***p ≤ 0.001). Binomial probabilities (*bp*) for the number of observed correct identifications are above chance level (***p ≤ 0.001).

The number of correct responses for the lineups with three BOs (23/24 = 96%, binomial probability, bp, for that result or higher by chance = 1.73 × 10^-10^), five BOs (14/25 = 56%, bp = 7.63 × 10^-5^), and eight BOs (11/24 = 46%, bp = 6.03 × 10^-5^) were all above chance level (see **Figure 1**). In order to test the difference in performance between the lineup tests and controlling for the different chance levels we calculated odds ratios. The expected (chance-level) odds for correct responses were 1/2, 1/4, and 1/7 for the conditions with three, five, and eight BOs, respectively, while the observed odds were 23/1, 14/11, and 11/13, respectively.

The expected odds ratio when comparing the condition with three BOs with the condition with five BOs was (1/2)/(1/4) = 2 while the observed odds ratio was (23/1)/(14/11) = 18.071. The standard error for the natural logarithm of the odds ratio can be calculated with the formula (Bland and Altman, 2000): square root (1/23 + 1/1 + 1/14 + 1/11) = 1.098. The difference between the observed and the expected odds ratio can be re-calculated into a *z*-score with the formula: (LN(18.071) – LN(2))/1.098 = 2.00. A *z*-score of 2.00 corresponds to a probability (two-tailed) of 4.5%, which means that the probability of getting such a large, or larger, difference between observed and expected odds ratios (based on the difference in chance levels) as in the present case by chance is 0.045.

When comparing the odds for correct responses in the conditions with three and eight BOs the expected odds ratio was (1/2)/(1/7) = 3.5 while the observed odds ratio was (23/1)/(11/13) = 27.182 (*z* = 1.862, *p* = 0.063, for the difference) and when comparing the conditions with five and eight BOs the expected ratio was (1/4)/(1/7) = 1.75 and the observed ratio (14/11)/(11/13) = 1.504 (*z* = -0.263, *p* = 0.793, for the difference).

#### Participants’ Stress and Anxiety Levels

Several studies in eyewitness have shown that stress and anxiety may impair the identification performance (e.g., Houston et al., 2013). In order to assess these variables and verify if they influence the nosewitness identification, prior to the presentation of each video, participant rated their perceived stress on a visual analog scale and rated their state anxiety using STAI-S (Silva and Spielberger, 2007). The stress measurement was repeated after the lineup test. Stress levels decreased from the beginning (*M* = 29.92, *SD* = 26.48) to the end of the experiment [*M* = 25.04, *SD* = 25.76; *t*(72) = 2.08, *p* = 0.04]. The correlation between stress levels and performance were low and not significant [*r_pb_*(71) = -0.21, *p* > 0.05]. Anxiety levels increased from the beginning (*M* = 52.55, *SD* = 3.81) to the end of the experiment [*M* = 53.55, *SD* = 4.38; *t*(72) = -2.32, *p* = 0.02]. State anxiety did not correlate with performance [*r_pb_*(71) = 0.02, *p* = 0.867].

In the 15-min delay between the witness session and lineup test, participants completed a questionnaire assessing trait anxiety (STAI-T, Silva and Spielberger, 2007), which did not correlate with later identification performance [*r_pb_*(71) = 0.17, *p* = 0.150].

#### Confidence of Identification

The literature in eyewitness identification often shows that confidence is a poor predictor of accuracy (Sporer et al., 1995; Krug, 2007). Early identification in lineup tests, however, may yield more encouraging results (Wixted et al., 2015). In our experiment, identification was positively correlated with participants’ confidence in their identification for the 5- and 8-BO lineup conditions [*r_pb_*(23) = 0.68, *p* < 0.001; *r_pb_*(22) = 0.50, *p* = 0.01, respectively]. However, this was not verified for the 3-BO lineup condition [*r_pb_*(22) = 0.10, *p* = 0.642], probably due to a ceiling effect as all but one participant identified the culprit in this condition.

#### Sex Differences

The chi-square tests did not show statistically significant differences between women and men for any lineup size condition (all *p*s > 0.05).

## Experiment 2

As noted, studies on odor memory revealed surprisingly little forgetting over time (Lawless, 1978; Murphy et al., 1991; Saive et al., 2014). This seemed to be valid for longer RIs (days, weeks, and 1 year; Engen and Ross, 1973; Lawless and Cain, 1975; Olsson et al., 2009) as well as for shorter ones (seconds to minutes; Engen et al., 1973; Jones et al., 1975; Jehl et al., 1994). More recent studies have rebutted these results by showing substantial forgetting over time in line with e.g., memory for faces (Cornell Kärnekull et al., 2015). Odors that are unfamiliar (and non-identifiable by name as is the case with BOs) are typically more difficult to retrieve, but are forgotten at the same rate as familiar and identifiable odors (Olsson et al., 2009; Cornell Kärnekull et al., 2015). Using the same general nosewitness paradigm as in Experiment 1, memory for BOs as a function of RI was tested below for the first time.

### Method

#### Body Odor Samples

Body odor samples were collected from the armpits of 25 healthy male students from University of Aveiro, aged between 18 and 25 years (*M* = 21.52, *SD* = 2.28), while in class for 4 h (non-stressful period). The restrictions given to the donors and the procedures of BO sampling were the same as in Experiment 1.

#### Participants

Forty students (20 males and 20 females aged between 18 and 31 years, *M* = 21.95, *SD* = 2.59) from University of Aveiro volunteered to participate. The participants did not suffer from any mental, neurological, metabolic, or respiratory diseases and were medication free. All the behavioral restrictions to reduce exogenous odors were the same as in Experiment 1. Participants and donors signed an informed consent and when applicable were rewarded with course credits.

#### Design and Procedure

In a between-subject design, participants were randomly assigned to one of the two experimental conditions: a short retention interval (SRI; 15 min, *n* = 20) and a long retention interval (LRI; 1 week, *n* = 20). Each participant viewed a video clip of a crime involving a man (culprit) and a woman. The videos, instructions, procedure and scales were the same used in Experiment 1. The difference was in the LRI condition where participants did the lineup test 1 week after the witness session.

The counterbalancing of the BOs was made assuring that the samples were thawed and refrozen the same number of times in each condition and that the culprit BO was presented in each of the five positions. Similar to Experiment 1, the targets were not used as foils, but the BO samples were the same in the SRI and LRI, in order to ensure that the differences between conditions were not due to the presentation of different BOs.

### Results and Discussion

#### Nosewitness Experience

As in Experiment 1, the ratings of the crime videos indicated that the crimes were experienced as highly vivid (*M* = 7.05, *SD* = 1.64; *M* = 7.00, *SD* = 0.92, for video #1 and #2, respectively), arousing (*M* = 6.90, *SD* = 1.71 *M* = 6.70, *SD* = 1.63, for video #1 and #2, respectively) and unpleasant (*M* = 1.80, *SD* = 1.11; *M* = 2.10, *SD* = 1.02, for video #1 and #2, respectively). Independent samples *t-*tests of the ratings of crime videos indicated no statistically significant difference in the evaluation of the films (*p* > 0.05). They will therefore not be a factor in the following analyses.

#### Lineup Identification Performance

**FIGURE 2:**
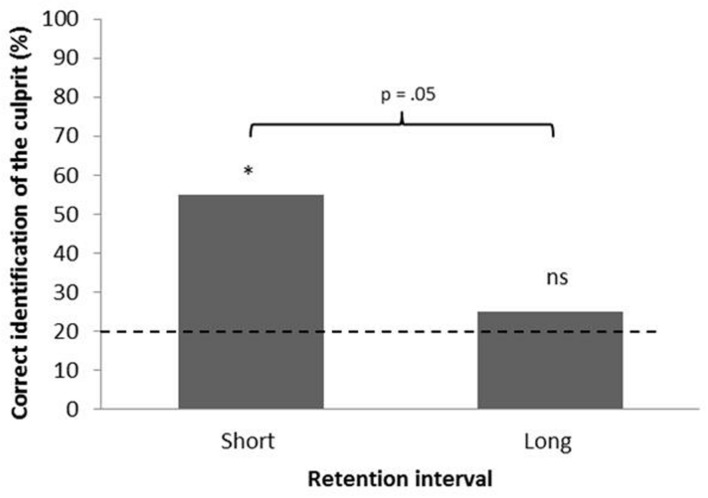
**Percentage of participants correctly identifying the culprit odor in the two RI conditions.** Dashed line represents chance level (20%). *p ≤ 0.05. Binomial probability (*bp*) indicates that the performance in the short retention condition is significantly above chance.

The number of correct responses for the SRI (11 correct responses = 55%, binomial probability, bp = 5.63 × 10^-4^) was significantly above chance level (20%), whereas performance for the LRI (5 correct responses = 25%, binomial probability, bp = 0.370) was not (see **Figure 2**).

A chi-square analysis was performed and the effect was marginally significant, indicating increased forgetting over time [χ^2^(1) = 3.75, *p* = 0.053; Cramer’s φ = 0.31].

#### Participants’ Stress and Anxiety Levels

As in Experiment 1, participants rated their perceived stress on a 100 mm visual analog scale as well as their state anxiety using STAI-S (Silva and Spielberger, 2007) prior to the presentation of each video.

Stress levels for SRI and LRI increased insignificantly (*p*s > 0.05) from the beginning (*M* = 25.65, *SD* = 19.22; *M* = 22.65, *SD* = 15.81, respectively) to the end of the experiment (*M* = 28.70, *SD* = 21.49; *M* = 24.65, *SD* = 23.49, respectively). Moreover, there was no correlation between stress levels and performance [*r_pb_*(38) = 0.04, *p* = 0.806].

Anxiety levels for SRI and LRI increased insignificantly from the beginning (*M* = 34.05, *SD* = 6.50, *M* = 32.00, *SD* = 8.21, respectively) to the end of the experiment (*M* = 35.20, *SD* = 8.22; *M* = 32.65, *SD* = 10.29, respectively). There was also no significant correlation between state anxiety and performance [*r_pb_*(38) = 0.09, *p* = 0.581].

Finally, concerning the trait anxiety (STAI-T, Silva and Spielberger, 2007), results showed a statistically insignificant negative correlation [*r_pb_*(38) = -0.07, *p* = 0.668] with performance.

#### Confidence of Identification

There was a statistical tendency for a positive correlation between accuracy of identification and level of confidence [*r_pb_*(38) = 0.29, *p* = 0.07] across the two conditions.

#### Sex Differences

Chi-square tests did not show significant differences in performance between women and men (*ps* > 0.05).

## General Discussion

Lineup size and the RI between inspection and identification have both been shown to affect eyewitness accuracy (e.g., Leach et al., 2009). In the current study, we investigated these factors for nosewitness identification.

In Experiment 1, the results corroborated our hypothesis and showed a higher relative performance rate (i.e., a significant difference between observed and expected odds ratios) for smaller lineups (three BOs). The intrinsic difficulty of discriminating odors (Olsson and Cain, 2000) and processing them in working memory (Jönsson et al., 2011) are possible reasons for this observation. Our results are similar to those of eye- and earwitness studies in that identification decreases as the lineup size increases (Yarmey, 1994, 2007; Meissner et al., 2005).

In our second experiment, we investigated the rate of forgetting BOs over time by using two RIs of 15 min and 1-week. In the short RI, the results from Experiment 1 were replicated (for the 5-BOs lineup trials), showing correct identifications between 50 and 60% of the trials. In the longer RI, performance was lower, as can be predicted from research involving a variety of memory tasks including eyewitness lineup identification (Deffenbacher et al., 2008). Several studies on earwitness identification used SRIs, such as 24 h or less (Philippon et al., 2007; Yarmey, 2007) and some have found little or no decrease in identification accuracy over a 24-h period (e.g., Saslove and Yarmey, 1980), whereas others have shown significant forgetting. Clifford et al. (1981), for instance, found that identification declined from 55% correct identification at 10 min to 32% after 24 h, with chance level being 5%. Studies that have used LRIs also show mixed results. For example, Bull and Clifford (1984) found that voice identification declined over l-week, 2-week, and 3-week retention periods from rates of 50 and 43% to chance level of 9%, respectively.

As noted above, early investigations indicated that odor memory was unique for its slow forgetting (e.g., Engen and Ross, 1973) and related that to smell’s privileged connection with limbic areas (Herz, 2005). This idea is consonant with some studies indicating that olfactory stimuli can cue autobiographical memories in a more vivid and emotional way than cues from other sensory modalities (Chu and Downes, 2000; Larsson and Willander, 2009). This would suggest that BO identification may not suffer a great impairment across RIs compared to visual or auditory cues. However, as indicated above, the impairment has been demonstrated in recent investigations in which odors show a similar rate of forgetting across RIs (e.g., Cornell Kärnekull et al., 2015) on par with studies on voices (e.g., Legge et al., 1984) and visual forms (e.g., Lawless, 1978). Notably, in the present study, levels of performance observed after 1 week were only insignificantly higher than chance. Thus, our results are consonant with the literature on forgetting, in which a LRI impairs identification.

The identification rate in the two conditions of the current study when using lineup sizes of 5 were as noted 56 and 55%. In the previous study (Alho et al., 2015, Experiment 1) the identification rate was considerably higher, 68%. One possible confounder in comparing the performance between experiments is the discriminability of target and foils. In a recent study testing the discriminability of BOs (Allen et al., 2015), BOs unknown to the participants were found to be discriminable in around 2/3 of the cases with the chance level being 1/3. If this level of performance would set the limit for how well one can perform in a nosewitness test, the observed levels (between 55 and 68% correct) are unexpectedly high. It is possible that the emotional encoding condition used in the nosewitness experiments actually improves the identification performance. Indeed, in the Alho et al. (2015) experiments, the emotional content of the videos during encoding boosted identification performance of the culprit BO. Interestingly, this is contrary to what typically happens in eyewitness studies (e.g., Deffenbacher et al., 2004; Houston et al., 2013).

Witness testimony in the judicial system relies solely on eyewitness and earwitness memory (e.g., The Innocence Project, 2015). With this background we investigate how olfaction can be an asset in criminal investigations. Some observations already testify to that end – The Cognitive Interview already considers olfactory information by asking the victim/witness about any odor that she/he can remember (e.g., Brunel et al., 2013). Moreover, some reports indicate that testimony about the culprit’s odor given by the victims has indeed been important for their identification and conviction (e.g., Doege, 1992). In the present study we replicate previous results found in our laboratory (Alho et al., 2015) showing that humans can indeed remember the body odor of unknown individuals in a forensic set up. Memory performance in a forced-choice test is substantial although far from perfect. Alho et al. (2015, Experiment 2) used a target present/target absent (TP/TA) design similar to eyewitness procedures. Again, participants could readily identify a present culprit. However, false alarm rates for target-absent lineups were substantial. With this in mind, in the present studies we decided to solely rely in target-present lineups. One may argue that, if olfaction is prone to false alarms, the use of nosewitness procedures in real life may be undermined. However, although target-absent lineups do not seem to be useful in the laboratory, future studies should be replicated in ecological settings in order to attest the reliability of nosewitness identification with this type of lineups. Moreover, both laboratory and real life experiments should also compare eye- and nosewitness performance in TP/TA studies that may provide important inputs in criminal investigations.

In these first experiments, we controlled several variables that could interfere with the participants’ performance. For example, donors were given behavioral restrictions in order to allow the use of endogenous BO samples. However, in daily life BOs are influenced by hygiene habits, scented products, food and beverage. Allen et al. (2015) showed that participants could still match a scented BO to an unscented version of the same BO, but that the scent made it more difficult. Future studies should investigate the effects of exogeneous odors on BO lineup identification.

In sum, this study shows that identification of BO in a forensic setting is possible and has characteristics in line with witness identification through other modalities, altogether meriting further investigation in this new field. Olfactory memory may turn out to be an interesting forensic tool, either in the identification of culprits or in the recollection of event details. Future avenues of research should entail the effects of emotion during encoding and further testing using the target-absent/target-present approach.

## Author Contributions

LA, SS, CS, and MO designed the experiments; LA, EP, LC, and JF collected the samples (BOs) and collected the data under the supervision of SS and CS; LA, EP, LC, and KS analyzed the data in collaboration with SS, CS, and MO; LA wrote the manuscript with input from all the coauthors.

## Conflict of Interest Statement

The authors declare that the research was conducted in the absence of any commercial or financial relationships that could be construed as a potential conflict of interest.
